# PHYSICAL ACTIVITY, PURPOSE, AND COGNITION: TESTING POSSIBLE MEDIATION PATHWAYS WITH CROSS-LAGGED PANEL ANALYSIS

**DOI:** 10.1093/geroni/igae098.3687

**Published:** 2024-12-31

**Authors:** Farima Sadeqi, Ryan Best

**Affiliations:** West Virginia University, Morgantown, West Virginia, United States; West Virginia University, Morgantown, West Virginia, United States

## Abstract

Numerous studies have emphasized the cognitive benefits older adults experience from engaging in regular physical activity. Previous research has largely highlighted the physiological mechanisms of this relationship, like improvements in cardiovascular health and brain structure, but recent work has begun investigating possible psychological pathways. Prior work from the authors supported the hypothesis that purpose in life acts as a mediator between physical activity and cognitive function, though research suggests a bidirectional relationship between physical activity and purpose in life. Given the bidirectional nature of the relationship between these two variables, the current work tests the possibility that physical activity could also act as the mediator in a single model using a structural equation modeling framework. In a sample of 2960 adults aged 33 to 92 collected through two waves of the Midlife in the United States (MIDUS) study, we examined two potential mediation pathways in a cross-lagged panel model where purpose in life mediates the effect of physical activity on cognition and vice versa. Two separate models test mediation pathways on executive functioning and episodic memory. Results show that purpose in life acted as a significant mediator between physical activity and both cognitive abilities (p <.021), while the indirect effects of physical activity between purpose in life and both cognitive abilities were not significant (p >.67). Findings from this study suggest that among physical activities with similar cardiovascular benefits, the one that also nurtures individuals’ purpose in life and boosts psychological well-being should be prioritized.

